# Supplementary material for: Cancer-Associated Fibroblasts Influence Survival in Pleural Mesothelioma: Digital Gene Expression Analysis and Supervised Machine Learning Model
Source: Int J Mol Sci. 2023 Aug 4;24(15):12426. doi: 10.3390/ijms241512426 (PMC10419996; doi:10.3390/ijms241512426)
Supplement: Supplementary file 1 [file ijms-24-12426-s001.zip › ijms-2490727-supplementary.pdf]

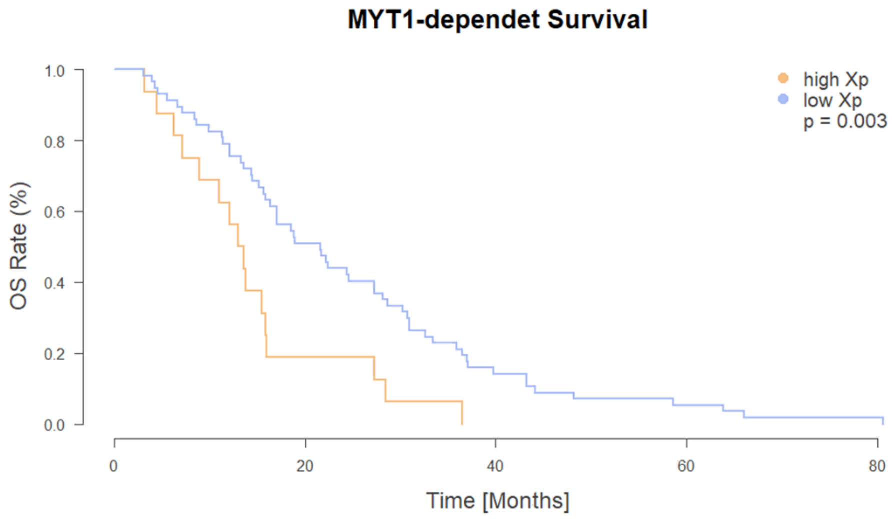

Figure S1. Kaplan-Meier curves for MYT1 expression demonstrated better OS with lower expression.

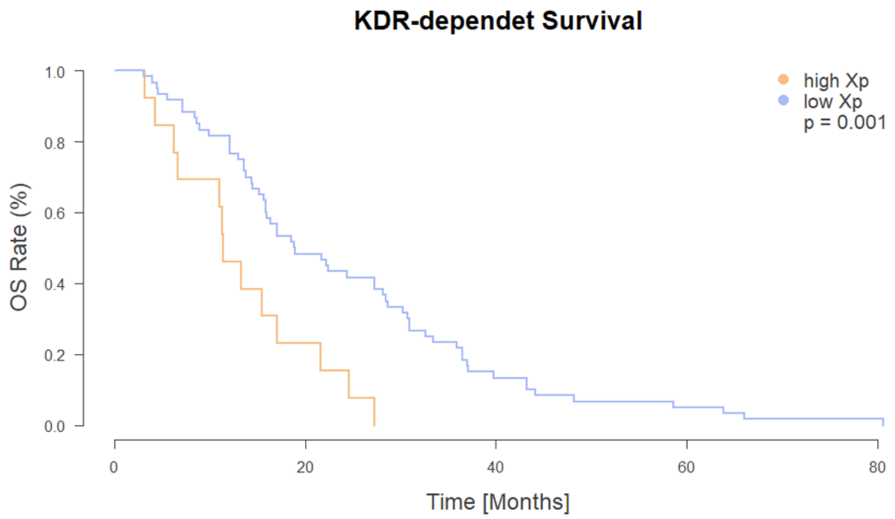

Figure S2. Kaplan-Meier curves for KDR expression demonstrated better OS with lower expression.

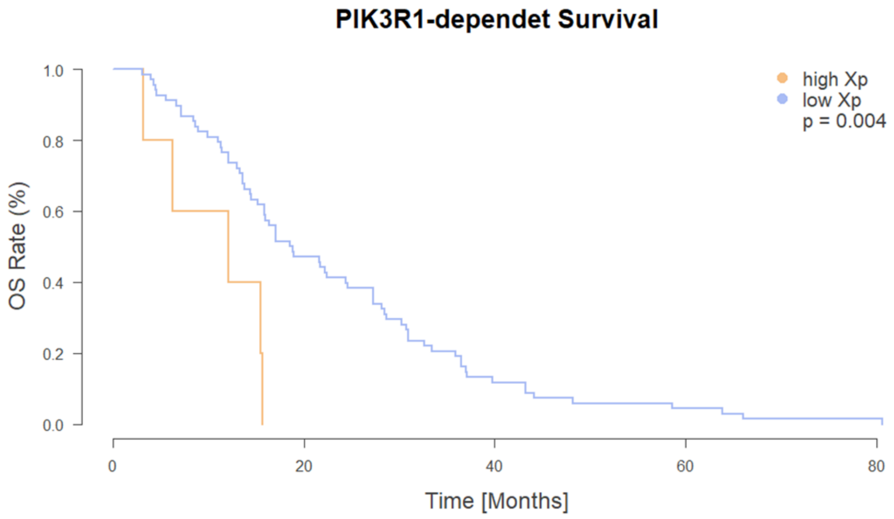

Figure S3. Kaplan-Meier curves for PIK3R1 expression demonstrated better OS with lower expression.

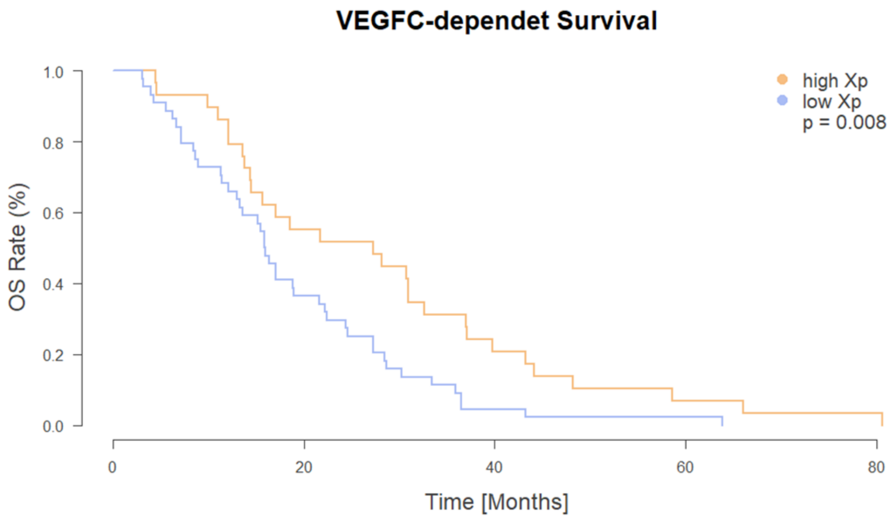

Figure S4. Kaplan-Meier curves for VEGFC expression demonstrated better OS with higher expression.

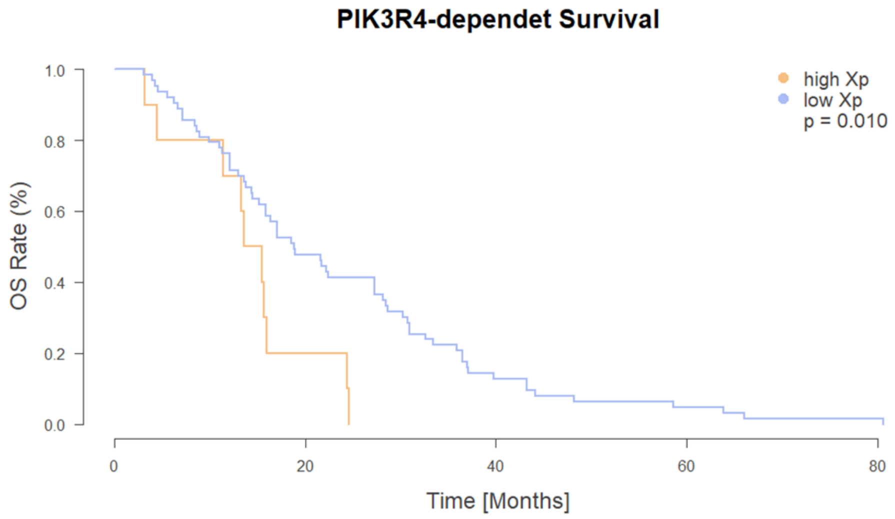

Figure S5. Kaplan-Meier curves for PIK3R4 expression demonstrated better OS with lower expression.

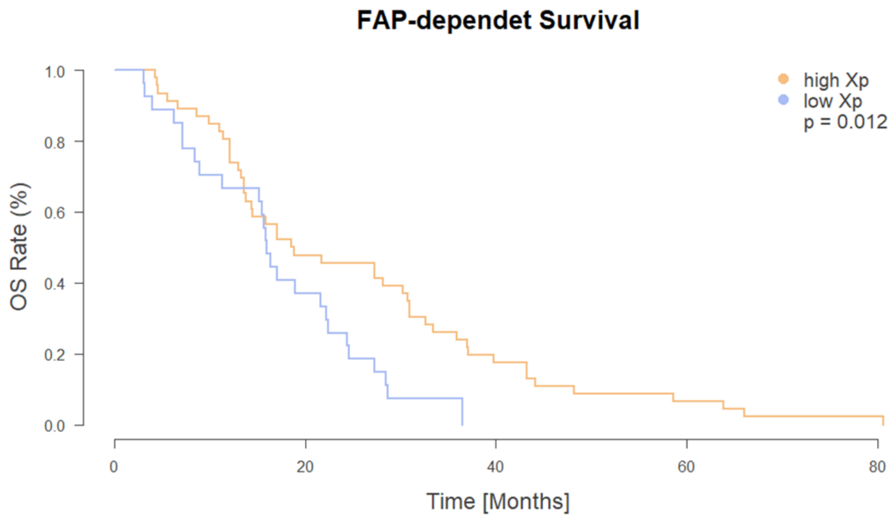

Figure S6. Kaplan-Meier curves for FAP expression demonstrated better OS with higher expression.

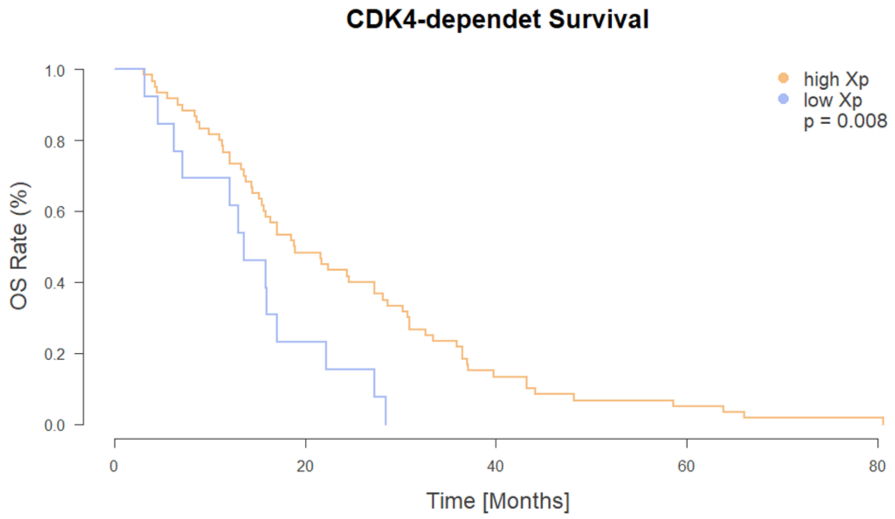

Figure S7. Kaplan-Meier curves for CDK4 expression demonstrated better OS with higher expression.

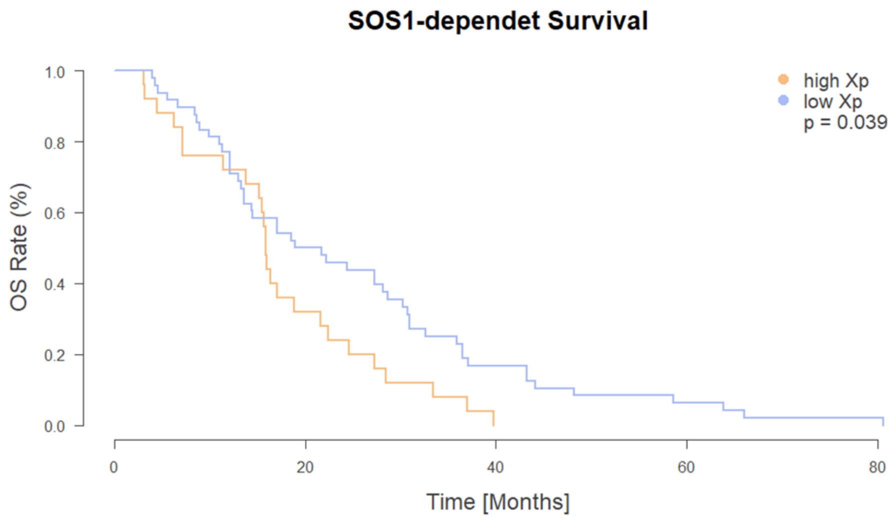

Figure S8. Kaplan-Meier curves for SOS-1 expression demonstrated better OS with lower expression.

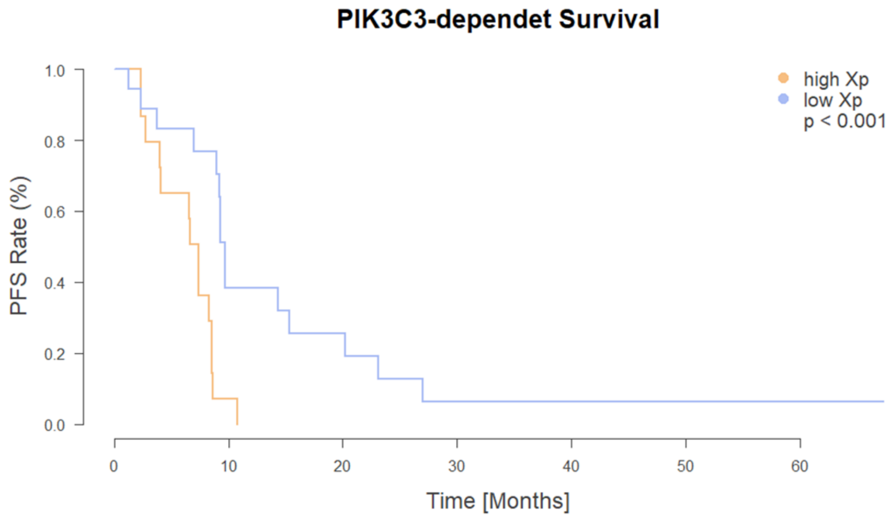

Figure S9. Kaplan-Meier curves for PIK3C3 expression demonstrated better PFS with lower expression.

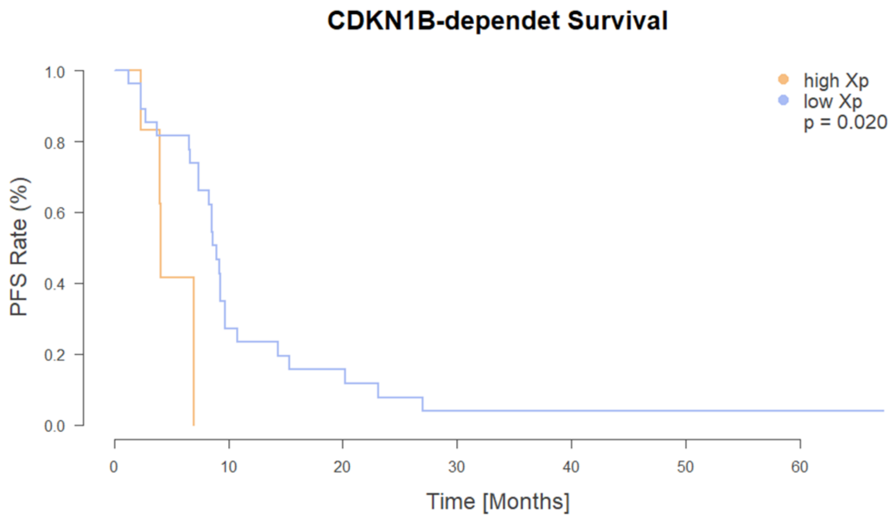

Figure S10. Kaplan-Meier curves for CDKN1B expression demonstrated better PFS with lower expression.

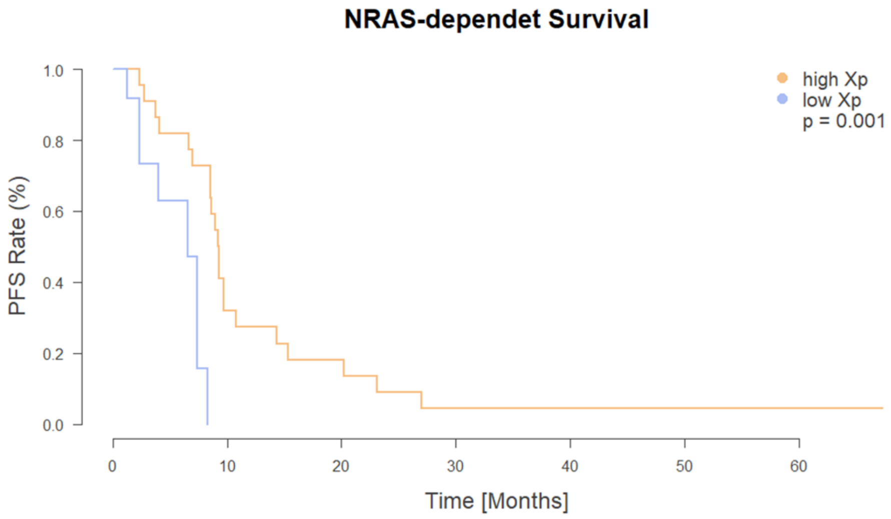

Figure S11. Kaplan-Meier curves for NRAS expression demonstrated better PFS with higher expression.

Supplementary Table S1. Significant marker found for overall survival (OS) and progression-free survival (PFS). HR = Hazard Ratio; NA = not available; DSR = desmoplastic stromal reaction

**Overall Survival**			
**Marker**	**Median OS (95% CI)**	**HR (95% CI)**	**p-Value**
Appearence of DSR	15.9 (13.8 - 27.3) vs. 18.7 (14.4 - 28.2)	1.04 (0.59 - 1.58)	0.8730
Amount of DSR	15.9 (17.1 - 44.2) vs. 29.2 (13.3 - 44.2)	1.21 (0.71 - 3.00	0.6080
High DSR vs. Others	29.2 (17.1 - 44.2) vs. 15.9 (11.4 - 27.3)	1.75 (1.02 - 3.00)	0.0440
FAP IHC	16.0 (15.2 - 30.3) vs. 17.1 (13.6 - 24.6)	1.04 (0.69 - 1.88)	0.0806
FAP mRNA	18.7 (14.4 – 30.9) vs. 16.0 (15.2 – 22.4)	1.93 (1.15 – 3.24)	0.0120
CDK4	18.9 (15.6 – 28.2) vs. 13.6 (7.2 – NA)	2.31 (1.22 – 4.35)	0.0080
KDR	11.4 (6.7 - NA) vs. 18.9 (15.9 - 28.5)	0.36 (0.19 - 0.68)	0.0010
MYT1	13.3 (8.9 – 27.3) vs. 21.6 (16.4 – 28.2)	0.42 (0.24 - 0.76)	0.0030
PIK3R1	12.1 (6.2 - NA) vs. 18.7 (15.9 - 27.3)	0.27 (0.10 - 0.69)	0.0040
PIK3R4	14.5 (14.5 - NA) vs. 18.9 (15.9 - 28.2)	0.42 (0.21 - 0.85)	0.0100
SOS1	15.9 (15.2 – 22.4) vs. 20.4 (14.4 – 30.3)	0.59 (0.35 – 0.98)	0.0390
VEGFC	27.3 (15.6 – 37.0) vs. 15.9 (13.3 – 22.2)	1.93 (1.18 – 3.18)	0.0080
**Progression-free Survival**			
**Marker**	**Median PFS (95% CI)**	**HR (95% CI)**	**p-Value**
Appearence of DSR	9.7 (4.1 - NA) vs. 8.5 (7.4 - 9.3)	1.26 (0.51- 3.1)	0.5970
Amount of DSR	8.6 (6.7 - 9.7) vs. 8.9 (7.0 - NA)	1.38 (0.33 - 1.59)	0.8730
FAP IHC	9.7 (6.5 - NA) vs. 7.39 (6.7 - 9.3)	1.47 (0.66 - 3.25)	0.8070
CDKN1B	4.1 (3.9 – NA) vs. 8.9 (8.3 – 10.8)	0.25 (0.07 - 0.88)	0.0200
NRAS	9.3 (8.5 – 14.3) vs. 6.5 (4.0 – NA)	5.23 (1.74 – 15.72)	0.0010
PIK3C3	7.4 (4.1 –8.6) vs. 9.7 (9.2 – 23.1)	0.24 (0.10 - 0.59)	p<0.001

Supplementary Table S2. Overview of all calculated p-values related to OS and gene expression.

Gene	Likelyhood Ratio Test	Score (logrank) Test	Wald Test
ABCB1 dependent Overall Survival	0.2300	0.2100	0.2100
ACTA2 dependent Overall Survival	0.6500	0.6500	0.6500
AKT1 dependent Overall Survival	0.5800	0.5800	0.5800
ARAF dependent Overall Survival	0.0760	0.0650	0.0650
ATG14 dependent Overall Survival	0.4500	0.4400	0.4400
BECN1 dependent Overall Survival	0.3700	0.3500	0.3500
BRAF dependent Overall Survival	0.6800	0.6700	0.6700
CCNA2 dependent Overall Survival	0.6300	0.6400	0.6400
CCND1 dependent Overall Survival	0.0410	0.0600	0.0610
CCNE1 dependent Overall Survival	0.7700	0.7700	0.7700
CD274 dependent Overall Survival	0.5000	0.4800	0.4800
CD44 dependent Overall Survival	0.3600	0.3700	0.3700
CD47 dependent Overall Survival	0.0760	0.0610	0.0600
CDK1 dependent Overall Survival	0.6300	0.6300	0.6300
CDK2 dependent Overall Survival	0.4500	0.4300	0.4300
CDK4 dependent Overall Survival	0.0300	0.0360	0.0360
CDK6 dependent Overall Survival	0.9300	0.9300	0.9300
CDKN1A dependent Overall Survival	0.3900	0.4000	0.4000
CDKN1B dependent Overall Survival	0.5000	0.4800	0.4900
CDKN2A dependent Overall Survival	0.7100	0.7000	0.7000
CHRM3 dependent Overall Survival	0.1800	0.1400	0.1400
CTNNB1 dependent Overall Survival	0.2800	0.2900	0.2900
EGF dependent Overall Survival	0.9000	0.9000	0.9000
EGFR dependent Overall Survival	0.8200	0.8300	0.8300
FAP dependent Overall Survival	0.0210	0.0340	0.0380
FGF7 dependent Overall Survival	0.1600	0.1300	0.1400
FLT1 dependent Overall Survival	0.5600	0.5700	0.5700
FLT4 dependent Overall Survival	0.6200	0.6300	0.6300
FN1 dependent Overall Survival	0.1400	0.1600	0.1600
FZD10 dependent Overall Survival	0.2400	0.1900	0.1900
FZD2 dependent Overall Survival	0.4500	0.4600	0.4600
FZD5 dependent Overall Survival	0.0840	0.0590	0.0610
HGF dependent Overall Survival	0.9200	0.9200	0.9200
HRAS dependent Overall Survival	0.8800	0.8800	0.8800
IGF1 dependent Overall Survival	0.4400	0.4500	0.4500
KDR dependent Overall Survival	0.0120	0.0057	0.0067

KRAS dependent Overall Survival	0.7200	0.7200	0.7200
MAP2K1 dependent Overall Survival	0.7900	0.7900	0.7900
MAP2K2 dependent Overall Survival	0.4600	0.4500	0.4500
MAPK1 dependent Overall Survival	0.9400	0.9400	0.9400
MDM2 dependent Overall Survival	0.7800	0.7800	0.7800
MET dependent Overall Survival	0.5400	0.5300	0.5300
MTOR dependent Overall Survival	0.4800	0.4800	0.4800
MYC dependent Overall Survival	0.2000	0.1700	0.1800
MYT1 dependent Overall Survival	0.0087	0.0015	0.0022
NF1 dependent Overall Survival	0.7300	0.7300	0.7300
NRAS dependent Overall Survival	0.3800	0.3800	0.3800
PDGFB dependent Overall Survival	0.8500	0.8500	0.8500
PIK3C3 dependent Overall Survival	0.1100	0.1000	0.1000
PIK3CA dependent Overall Survival	0.3900	0.3800	0.3800
PIK3R1 dependent Overall Survival	0.0130	0.0059	0.0062
PIK3R4 dependent Overall Survival	0.0270	0.0220	0.0230
PIK3R5 dependent Overall Survival	0.9700	0.9700	0.9700
PTEN dependent Overall Survival	0.1900	0.1800	0.1800
RAF1 dependent Overall Survival	0.5500	0.5400	0.5400
RICTOR dependent Overall Survival	0.2900	0.2800	0.2800
RPS6KB1 dependent Overall Survival	0.0460	0.0510	0.0510
RPTOR dependent Overall Survival	0.2900	0.2700	0.2700
SMAD2 dependent Overall Survival	0.2000	0.2000	0.2000
SMAD4 dependent Overall Survival	0.7200	0.7200	0.7200
SMAD7 dependent Overall Survival	0.6700	0.6700	0.6700
SOS1 dependent Overall Survival	0.0460	0.0390	0.0390
SOS2 dependent Overall Survival	0.1900	0.1800	0.1800
TGFA dependent Overall Survival	0.3600	0.3300	0.3300
TGFB1 dependent Overall Survival	0.6700	0.6700	0.6700
TGFBR1 dependent Overall Survival	0.2900	0.3100	0.3100
TGFBR2 dependent Overall Survival	0.3800	0.3600	0.3600
TP53 dependent Overall Survival	0.4000	0.4000	0.4000
VEGFA dependent Overall Survival	0.9800	0.9800	0.9800

VEGFC dependent Overall Survival	0.0110	0.0160	0.0170
WEE1 dependent Overall Survival	0.9000	0.9000	0.9000
WNT1 dependent Overall Survival	0.0960	0.0520	0.0590
WNT2 dependent Overall Survival	0.4400	0.4300	0.4300
WNT3 dependent Overall Survival	0.4700	0.4500	0.4500

Supplementary Table S3. Overview of all the calculated p-values related to PFS and gene expression.

Gene	Likelyhood Ratio Test	Score (logrank) Test	Wald Test
ABCB1 dependent Progression Free Survival	0.8600	0.8600	0.8600
ACTA2 dependent Progression Free Survival	0.7900	0.7900	0.7900
AKT1 dependent Progression Free Survival	0.9400	0.9400	0.9400
ARAF dependent Progression Free Survival	0.4800	0.4500	0.4500
ATG14 dependent Progression Free Survival	0.4300	0.4100	0.4100
BECN1 dependent Progression Free Survival	0.0880	0.0690	0.0690
BRAF dependent Progression Free Survival	0.7600	0.7500	0.7500
CCNA2 dependent Progression Free Survival	0.4600	0.4700	0.4700
CCND1 dependent Progression Free Survival	0.2200	0.2500	0.2500
CCNE1 dependent Progression Free Survival	0.3000	0.2900	0.2900
CD274 dependent Progression Free Survival	0.5600	0.5400	0.5400
CD44 dependent Progression Free Survival	0.5900	0.6000	0.6000
CD47 dependent Progression Free Survival	0.0840	0.0590	0.0590
CDK1 dependent Progression Free Survival	0.7900	0.7900	0.7900
CDK2 dependent Progression Free Survival	0.2000	0.1900	0.1900
CDK4 dependent Progression Free Survival	0.4900	0.5000	0.5000
CDK6 dependent Progression Free Survival	0.8200	0.8200	0.8200
CDKN1A dependent Progression Free Survival	0.5900	0.6000	0.6000
CDKN1B dependent Progression Free Survival	0.0460	0.0210	0.0240
CDKN2A dependent Progression Free Survival	0.1300	0.0800	0.0910
CHRM3 dependent Progression Free Survival	0.5400	0.5100	0.5200
CTNNB1 dependent Progression Free Survival	0.3000	0.3100	0.3100
EGF dependent Progression Free Survival	0.2000	0.1500	0.1600
EGFR dependent Progression Free Survival	0.7100	0.7100	0.7100
FAP dependent Progression Free Survival	0.3500	0.3700	0.3700
FGF7 dependent Progression Free Survival	0.3900	0.3500	0.3600
FLT1 dependent Progression Free Survival	0.5800	0.5700	0.5700
FLT4 dependent Progression Free Survival	0.6700	0.6800	0.6800
FN1 dependent Progression Free Survival	0.1900	0.2100	0.2100
FZD10 dependent Progression Free Survival	0.6500	0.6300	0.6400

FZD2 dependent Progression Free Survival	0.3700	0.3900	0.3900
FZD5 dependent Progression Free Survival	0.5700	0.5600	0.5600
HGF dependent Progression Free Survival	0.9900	0.9900	0.9900
HRAS dependent Progression Free Survival	0.3500	0.3400	0.3500
IGF1 dependent Progression Free Survival	0.4200	0.4100	0.4100
KDR dependent Progression Free Survival	0.3600	0.3400	0.3400
KRAS dependent Progression Free Survival	0.6000	0.5900	0.5900
MAP2K1 dependent Progression Free Survival	0.5200	0.5100	0.5100
MAP2K2 dependent Progression Free Survival	0.2800	0.2600	0.2600
MAPK1 dependent Progression Free Survival	0.3800	0.3800	0.3800
MDM2 dependent Progression Free Survival	0.8200	0.8200	0.8200
MET dependent Progression Free Survival	0.8500	0.8500	0.8500
MTOR dependent Progression Free Survival	0.3100	0.3000	0.3000
MYC dependent Progression Free Survival	0.9500	0.9500	0.9500
MYT1 dependent Progression Free Survival	0.8500	0.8500	0.8500
NF1 dependent Progression Free Survival	0.2900	0.3000	0.3000
NRAS dependent Progression Free Survival	0.0160	0.0210	0.0200
PDGFB dependent Progression Free Survival	0.7400	0.7400	0.7400
PIK3C3 dependent Progression Free Survival	0.0140	0.0100	0.0100
PIK3CA dependent Progression Free Survival	0.1500	0.1600	0.1600
PIK3R1 dependent Progression Free Survival	0.9500	0.9500	0.9500
PIK3R4 dependent Progression Free Survival	0.3900	0.3700	0.3700
PIK3R5 dependent Progression Free Survival	0.7500	0.7500	0.7500
PTEN dependent Progression Free Survival	0.3100	0.3000	0.3000
RAF1 dependent Progression Free Survival	0.4600	0.4500	0.4500
RICTOR dependent Progression Free Survival	0.2700	0.2600	0.2600
RPS6KB1 dependent Progression Free Survival	0.8800	0.8800	0.8800
RPTOR dependent Progression Free Survival	0.3000	0.2800	0.2800
SMAD2 dependent Progression Free Survival	0.1800	0.1700	0.1700
SMAD4 dependent Progression Free Survival	0.1800	0.1700	0.1700
SMAD7 dependent Progression Free Survival	0.8900	0.8900	0.8900
SOS1 dependent Progression Free Survival	0.2600	0.2400	0.2400
SOS2 dependent Progression Free Survival	0.6900	0.6800	0.6800
TGFA dependent Progression Free Survival	0.6200	0.6000	0.6000

TGFB1 dependent Progression Free Survival	0.2900	0.3100	0.3200
TGFBR1 dependent Progression Free Survival	0.0460	0.0660	0.0700
TGFBR2 dependent Progression Free Survival	0.8000	0.8000	0.8000
TP53 dependent Progression Free Survival	0.6400	0.6400	0.6500
VEGFA dependent Progression Free Survival	0.9500	0.9500	0.9500
VEGFC dependent Progression Free Survival	0.3900	0.4100	0.4100
WEE1 dependent Progression Free Survival	0.6700	0.6800	0.6900
WNT1 dependent Progression Free Survival	0.8700	0.8700	0.8700
WNT2 dependent Progression Free Survival	0.7300	0.7300	0.7300
WNT3 dependent Progression Free Survival	0.1100	0.0690	0.0770

Supplementary Table S4. Targets of the respective pathways in the custom-designed codeset used for digital gene expression analysis.

**Cell cycle**		**PI3K signaling**		**MAPK**		**Wnt**		**Growth signaling**		**TGF-β**	**Fibroblasts**
CDK1	*CDKN2A*	*PIK3C3*	*AKT1*	*MAP2K1*	*SOS1*	*CTNNB1*	*FZD2*	*EGFR*	*KDR*	*TGFB1*	*ACTA2*
CDK2	*CDKN1B*	*ATG14*	*PIK3CA*	*KRAS*	*SOS2*	*WNT1*	*FZD5*	*TGFA*	*PDGFB*	*TGFBR1*	*FAP*
CDK4	*CDKN1A*	*PIK3R5*	*MTOR*	*MAPK1*	*NF1*	*WNT2*		*EGF*	*IGF1*	*TGFBR2*	*FN1*
CDK6	*CCND1*	*PIK3R4*	*PTEN*	*BRAF*	*MAP2K2*	*CD47*		*HGF*	*VEGFC*	*CD44*	
MDM2	*CCNA2*	*PIK3R1*	*RICTOR*	*CHRM3*	*RAF1*	*CD274*		*FGF1*	*MET*	*SMAD4*	
TP53	*CCNB3*	*PIK3CA*	*RPTOR*	*HRAS*		*ABCB1*		*VEGFA*		*SMAD2*	
WEE1	*CCNE1*	*RPS6KB1*		*NRAS*		*WNT3*		*FLT1*		*SMAD7*	
Myt1		*BECN1*		*ARAF*		*FZD10*		*FLT4*		*MYC*	
